# Short Term Outcome of Patients with Hematochezia and Normal Initial Colonoscopic Findings: Do They Really Need Further Screening?

**Published:** 2011-12-01

**Authors:** S A Taghavi, S Sha`bani, M Khademalhoseini, N Shabanipour, A Mehramiri, S Negahban, S Yahyazadeh, A Eshraghian

**Affiliations:** 1 Gastroenterology Research Centre, Shiraz University of Medical Sciences, Shiraz, Iran

**Keywords:** Hematochezia, Colonoscopy, Iran, Outcome

## Abstract

**Background:**

In a significant number of the patients with hematochezia, colonoscopy turns out to be normal and therefore is unable to determine the cause of bleeding. This study investigates outcomes and possible necessity for further work up in cases of hematochezia with normal colonoscopy.

**Methods:**

Ninety-seven patients with normal colonoscopy were followed for at least one year from the time of colonoscopy by regular visits and phone calls. Mortality and recurrent bleeding were recorded as primary end points. Those with recurrent or continued hematochezia were invited for a new visit and further work up.

**Results:**

Among the ninety seven patients, nine cases (9.3%) were lost at follow ups, 10 experienced rebleeding (10.3 %), and the remaining 78 (80.4 %) were apparently healthy and had no further complaints. There were two mortalities during the follow up, one due to gastric cancer and the other due to cerebrovascular accident.

**Conclusion:**

It is unusual for the cases of hematochezia with a normal initial colonoscopy to have recurrent bleeding as a result of a significant missed lesion in the colon.

## Introduction

Hematochezia, the passage of bright red or maroon blood from the rectum, usually originates from a source in lower parts of the gastrointestinal (GI) tract especially in a homodynamically stable patient.[1] The numerous aetiologies which may cause hematochezia can be grouped into several categories including anatomic (e.g. diverticulosis); vascular (e.g. angiodysplasia, ischemia and radiation-induced); inflammatory

(e.g. infectious and idiopathic inflammatory bowel diseases); neoplastic causes (e.g. polyp, carcinoma) and miscellaneous disorders (e.g. haemorrhoid and solitary ulcers ) and rare systemic causes (e.g. vitamin K deficiency and other coagulative disorders).[2][3][4]

There is an array of para-clinical tests which are routinely used to find the cause of bleeding; including colonoscopy, RBC scan, angiography and even surgical exploration in severe cases. [2]Ignoring the surgical interventions for obvious reasons, colonoscopy has the highest diagnostic yield among the above mentioned interventions. It is the selective tool for evaluation of the entire colon for neoplastic and non neoplastic sources of bleeding.[5][6] It should be noted that the most careful clinical assessment can be even unreliable both for determining the site of bleeding and for ruling out significant pathology.[7][8][9][10] As an example, the presence of haemorrhoids does not exclude a more proximal culprit lesion, and could even mask the concomitant bleeding of an early neoplasia.[9]

In a significant number of the patients with hematochezia, colonoscopy turns out to be normal and therefore is unable to determine the cause of bleeding. Most of these cases would stop bleeding spontaneously with no treatment but in rare cases rebleeding may occur inviting the physician for more intensive work up.[2][4] Some of these patients may have more serious gastrointestinal (GI) disorders, including vascular lesions, GISTs, Crohn‘s disease, intestinal tuberculosis (TB), ischemic colitis and malignancies,[3][4][11][12][13][14][15] which all are preferred to be distinguished in early stages of presentation.[4][11][12][13][14][15]

This study was designed to follow the cases of hematochezia with normal colonoscopy and to determine the prognosis as well as necessity of the further work up in such cases.

## Materials and Methods 

All patients aged 15 to 75 years old presenting with hematochezia between April 2006 and end of December 2006. All patients were referred to two university affiliated clinics of Shiraz University of Medical Sciences, Shiraz, Southern Iran. Complaining of excretion of red blood per rectum and a normal total colonoscopy were the criteria for the patients to be enrolled.

The exclusion criteria were past medical history of any GI disorder which may present with fresh rectal bleeding including diverticulosis; vascular abnormalities, radiation-induced pathologies; inflammatory bowel disease (IBD); neoplasm and other anorectal disorders such as haemorrhoid, ulcer as well as any colonoscopic findings which can produce fresh rectal bleeding. Ninety-seven patients with normal colonoscopy were included in then study.

**Fig. 1:**
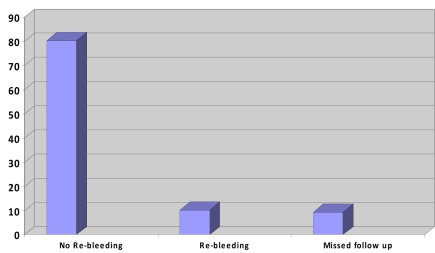
Rebleeding rate in the study group.

All patients were firstly visited by a gastroenterologist. On first visit, a questionnaire was filled for each patient during a face-to-face interview about the pattern, duration, amount of their rectal bleeding, their bowl habit and their past medical history of any gastro intestinal and non-gastrointestinal disorders. The same questionnaire was also completed on the follow up visit of those experiencing rebleeding (Figure 1).

Before the procedure, complete bowel preparation was performed for all patients. At least twelve to fifteen hours prior to procedure, patients were instructed to drink 280 g of polyethylene glycol powder ( PIDROLAX, Sepidag Pharmacologic Company, Karaj, Iran) solved in four litter of water in fifteen minutes intervals. All colonoscopies were performed using an Olympus Q230 or Q240 videocolonope (Olympus Co., Tokyo, Japan), by a single expert gastroenterologist to eliminate any person-to-person variability. The completeness of colonoscopy was assured by visualization of terminal ileum.

Ninety-seven patients with normal colonoscopy were followed for at least one year period from the time of colonoscopy. The patients were either seen personally in the clinic every three months or, for those who did not attend their scheduled visits, followed by phone calls. Those with recurrence or continuation of hematochezia were invited for a new visit and further work up. On their visits, history and physical exam were performed again and colonoscopy was done. Data were collected and analyzed with SPSS software(Version 15, Chicago, IL, USA), using Student t and Chi Square tests.

This study was approved by constituted Ethics Committee of the Shiraz University of Medical Sciences within which the work was undertaken. It also conformed to the provisions of the Declaration of Helsinki (as revised in Edinburgh 2000).

## Results 

Baseline characteristics of the patients were outlined in Table 1. Among the 97 patients, 9 cases (9.3%) were lost at follow ups, 10 experienced rebleeding (10.3 %), and the remaining 78 (80.4 %) were apparently healthy and had no further complaints (p=0.014). Two patients expired during the follow up, one due to gastric cancer (who experienced rebleeding) and the other due to cerebro-vascular accident (no re-bleeding).

**Table 1:** Baseline characteristics of the patients.

Mean age (year)	48.34
Sex (male/female)	59/38
Mean hemoglobin level on presentation (mg/dL)	11.45
Mean blood pressure on presentation (mmHg)	12.13

Ten re-bleeding cases were invited for further work up; one presented with hematemesis and was subsequently diagnosed with gastric cancer, 9 underwent re-colonoscopy: two (20%) had high grade haemorrhoids that were referred to general surgeons for haemorrhoidectomy after which their bleeding stopped, one (10%) had mild proctitis (not present in the first colonoscopy), and the remaining six (60%) had normal colonoscopy. There were no statistically significant differences between the re-bleeder vs. non-rebleeder group in either mean age (p=0.167) or sex distribution (p=0.091).

## Discussion

Colonoscopy is useful in the investigation of patients with hematochezia but it is not successful in diagnosis of the aetiology of bleeding in a portion of patients, some of this group might have episodes of rebleeding but most of them do not experience more bleeding episodes.[10] Little information is available on the rate and predictive factors of rebleeding in those cases.[11] There is also little data to show whether a second look colonoscopy will be of any help.

In addition to the risk of rebleeding and possible associated complications, there is also a legitimate concern over possible missed etiologies, which may affect the patient’s future well being. [11][12][13][14][15]Our study, although small in size, clearly showed that majority of these patients (80.4 %) had no further bleeding episodes or complications, at least in short term follow ups. From those who completed the study only 2 of the patients were diagnosed with gastric cancer in one year follow up. In those patients who re-bled during follow ups, re-colonoscopy rarely showed the most feared neoplastic lesions but may elucidate the diagnosis and help the treatment in up to one –third of the patients.

Although any casual relation between gastric cancer and fresh rectal bleeding is questionable, this occurrence emphasized the need for detailed history taking as well as follows up in cases of hematochezia with undiagnosed aetiology.

In conclusion, neoplastic lesions of the large bowel are rare in patients with hematochezia and normal initial colonoscopy. According to the results of this study there is no need for further screening methods in patients with hematochazia at least during the first year. Re-colonoscopy is indicated in those with rebleeding.

## References

[1] Kasper DL, Brownwald E, Fauci AS, Hauser SL, Longo DL, Jamesone JL (2005). Harrison`s principle of internal medicine. 16ed..

[2] Misra SP, Misra V, Dwivedi M (2007). Ileoscopy in patients with ileocolonic tuberculosis.. World J Gastroenterol.

[3] Shah RJ, Fenoglio-Preiser C, Bleau BL, Giannella RA (2001). Usefulness of colonoscopy with biopsy in the evaluation of patients with chronic diarrhea.. Am J Gastroenterol.

[4] Segal WN, Greenberg PD, Rockey DC, Cello JP, McQuaid KR (1998). The outpatient evaluation of hematochezia.. Am J Gastroenterol.

[5] Imperiale TF, Wagner DR, Lin CY, Larkin GN, Rogge JD, Ransohoff DF (2000). Risk of advanced proximal neoplasms in asymptomatic adults according to the distal colorectal findings.. N Engl J Med.

[6] Lieberman DA, Weiss DG, Bond JH, Ahnen DJ, Garewal H, Chejfec G (2000). Use of colonoscopy to screen asymptomatic adults for colorectal cancer. Veterans Affairs Cooperative Study Group 380.. N Engl J Med.

[7] Helfand M, Marton KI, Zimmer-Gembeck MJ, Sox HC Jr (1997). History of visible rectal bleeding in a primary care population. Initial assessment and 10-year follow-up.. JAMA.

[8] Goulston KJ, Cook I, Dent OF (1986). How important is rectal bleeding in the diagnosis of bowel cancer and polyps?. Lancet.

[9] Mant A, Bokey EL, Chapuis PH, Killingback M, Hughes W, Koorey SG, Cook I, Goulston KJ, Dent OF (1989). Rectal bleeding. Do other symptoms aid in diagnosis?. Dis Colon Rectum.

[10] Graham DJ, Pritchard TJ, Bloom AD (1993). Colonoscopy for intermittent rectal bleeding: impact on patient management.. J Surg Res.

[11] de Bosset V, Froehlich F, Rey JP, Thorens J, Schneider C, Wietlisbach V, Vader JP, Burnand B, Muhlhaupt B, Fried M, Gonvers JJ (2002). Do explicit appropriateness criteria enhance the diagnostic yield of colonoscopy?. Endoscopy.

[12] Wong RF, Khosla R, Moore JH, Kuwada SK (2004). Consider colonoscopy for young patients with hematochezia.. J Fam Pract.

[13] Winawer S, Fletcher R, Rex D, Bond J, Burt R, Ferrucci J, Ganiats T, Levin T, Woolf S, Johnson D, Kirk L, Litin S, Simmang C (2003). Gastrointestinal Consortium Panel. Colorectal cancer screening and surveillance: Clinical guidelines and rationale-Update based on new evidence.. Gastroenterology.

[14] Raedle J, Trojan J, Brieger A, Weber N, Schäfer D, Plotz G, Staib-Sebler E, Kriener S, Lorenz M, Zeuzem S (2001). Bethesda guidelines: relation to microsatellite instability and MLH1 promoter methylation in patients with colorectal cancer.. Ann Intern Med.

[15] Zuckerman GR, Prakash C, Askin MP, Lewis BS (2000). AGA technical review on the evaluation and management of occult and obscure gastrointestinal bleeding.. Gastroenterology.

